# Sources and Importance of Mitochondrial NAD

**DOI:** 10.1093/geroni/igaa057.2623

**Published:** 2020-12-16

**Authors:** Joseph Baur, Timothy Luongo, Jared Eller, Mu-Jie Lu, Caroline Perry, Lulu Cambronne

**Affiliations:** 1 Perelman School of Medicine at the University of Pennsylvania, Philadelphia, Pennsylvania, United States; 2 University of Pennsylvania, Philadelphia, Pennsylvania, United States; 3 University of Texas at Austin, Austin, Texas, United States; 4 UT Austin, Austin, Texas, United States

## Abstract

Nicotinamide adenine dinucleotide (NAD) levels fall with age or disease, and rise with exercise or caloric restriction. Moreover, the demonstration that supplemental NAD precursors drive beneficial effects in rodent models has driven a resurgence in interest in the basic biology of this molecule. Although NAD is present in the mitochondrial matrix and critical to the function of the organelle, the source of mitochondrial NAD has been debated. We recently used isotopic labeling to demonstrate that direct uptake of intact NAD is one mechanism by which mitochondria are able to obtain this nucleotide. Here, we show that this activity is sufficient to restore respiratory capacity in NAD-deficient isolated mitochondria, and identify SLC25A51 as a carrier that can mediate the transport of NAD across mitochondrial membranes. Understanding the compartment-specific regulation of NAD will be crucial to understanding how cells and tissues adapt their metabolism to changes in NAD availability. Funding: DK098656 to J.A.B., GM126897 to L.A.C.

